# Incidence and Trends of Infection with Pathogens Transmitted Commonly Through Food — Foodborne Diseases Active Surveillance Network, 10 U.S. Sites, 1996–2012

**Published:** 2013-04-19

**Authors:** Debra Gilliss, Alicia B. Cronquist, Matthew Cartter, Melissa Tobin-D’Angelo, David Blythe, Kirk Smith, Sarah Lathrop, Shelley Zansky, Paul R. Cieslak, John Dunn, Kristin G. Holt, Susan Lance, Stacy M. Crim, Olga L. Henao, Mary Patrick, Patricia M. Griffin, Robert V. Tauxe

**Affiliations:** California Dept of Public Health; Colorado Dept of Public Health and Environment; Connecticut Dept of Public Health; Georgia Dept of Public Health; Maryland Dept of Health and Mental Hygiene; Minnesota Dept of Health; Univ of New Mexico; New York State Dept of Health; Oregon Health Authority; Tennessee Dept of Health; Food Safety and Inspection Svc, US Dept of Agriculture; Center for Food Safety and Applied Nutrition, Food and Drug Admin; Div of Foodborne, Waterborne, and Environmental Diseases, National Center for Emerging and Zoonotic Infectious Diseases, CDC

Foodborne diseases are an important public health problem in the United States. The Foodborne Diseases Active Surveillance Network* (FoodNet) conducts surveillance in 10 U.S. sites for all laboratory-confirmed infections caused by selected pathogens transmitted commonly through food to quantify them and monitor their incidence. This report summarizes 2012 preliminary surveillance data and describes trends since 1996. A total of 19,531 infections, 4,563 hospitalizations, and 68 deaths associated with foodborne diseases were reported in 2012. For most infections, incidence was highest among children aged <5 years; the percentage of persons hospitalized and the percentage who died were highest among persons aged ≥65 years. In 2012, compared with the 2006–2008 period, the overall incidence of infection† was unchanged, and the estimated incidence of infections caused by *Campylobacter* and *Vibrio* increased. These findings highlight the need for targeted action to address food safety gaps.

FoodNet conducts active, population-based surveillance for laboratory-confirmed infections caused by *Campylobacter*, *Cryptosporidium*, *Cyclospora*, *Listeria*, *Salmonella*, Shiga toxin–producing *Escherichia coli* (STEC) O157 and non-O157, *Shigella*, *Vibrio*, and *Yersinia* in 10 sites covering 15% of the U.S. population (48 million persons in 2011).§ FoodNet is a collaboration among CDC, 10 state health departments, the U.S. Department of Agriculture’s Food Safety and Inspection Service (USDA-FSIS), and the Food and Drug Administration (FDA). Hospitalizations occurring within 7 days of specimen collection date are recorded, as is the patient’s vital status at hospital discharge, or at 7 days after the specimen collection date if the patient was not hospitalized. All hospitalizations and deaths that occurred within a 7-day window are attributed to the infection. Surveillance for physician-diagnosed postdiarrheal hemolytic uremic syndrome (HUS), a complication of STEC infection characterized by renal failure, is conducted through a network of nephrologists and infection preventionists and by hospital discharge data review. This report includes 2011 HUS data for persons aged <18 years.

Incidence was calculated by dividing the number of laboratory-confirmed infections in 2012 by U.S. Census estimates of the surveillance population area for 2011.¶ A negative binomial model with 95% confidence intervals (CIs) was used to estimate changes in incidence from 2006–2008 to 2012 and from 1996–1998 to 2012 (1). The overall incidence of infection with six key pathogens for which >50% of illnesses are estimated to be foodborne (*Campylobacter*, *Listeria*, *Salmonella*, STEC O157, *Vibrio*, and *Yersinia*) was calculated (2). Trends were not assessed for *Cyclospora* because data were sparse, or for STEC non-O157 because of changes in diagnostic practices. For HUS, changes in incidence from 2006–2008 to 2011 were estimated.

## Incidence and Trends

In 2012, FoodNet identified 19,531 laboratory-confirmed cases of infection (Table 1). The number of infections and incidence per 100,000 population, by pathogen, were as follows: *Salmonella* (7,800; 16.42), *Campylobacter* (6,793; 14.30), *Shigella* (2,138; 4.50), *Cryptosporidium* (1,234; 2.60), STEC non-O157 (551; 1.16), STEC O157 (531; 1.12), *Vibrio* (193; 0.41), *Yersinia* (155; 0.33), *Listeria* (121; 0.25), and *Cyclospora* (15; 0.03). As usual, the highest reported incidence was among children aged <5 years for *Cryptosporidium* and the bacterial pathogens other than *Listeria* and *Vibrio*, for which the highest incidence was among persons aged ≥65 years (Table 2).

Among 6,984 (90%) serotyped *Salmonella* isolates, the top three serotypes were Enteritidis, 1,238 (18%); Typhimurium, 914 (13%); and Newport, 901 (13%). Among 183 (95%) *Vibrio* isolates with species information, 112 were *V. parahaemolyticus* (61%), 25 were *V. vulnificus* (14%), and 20 were *V. alginolyticus* (11%). Among 496 (90%) serogrouped STEC non-O157 isolates, the most common serogroups were O26 (27%), O103 (23%), and O111 (15%). Among 2,318 (34%) *Campylobacter* isolates with species information, 2,082 (90%) were *C. jejuni*, and 180 (8%) were *C. coli*.

**FIGURE 1:**
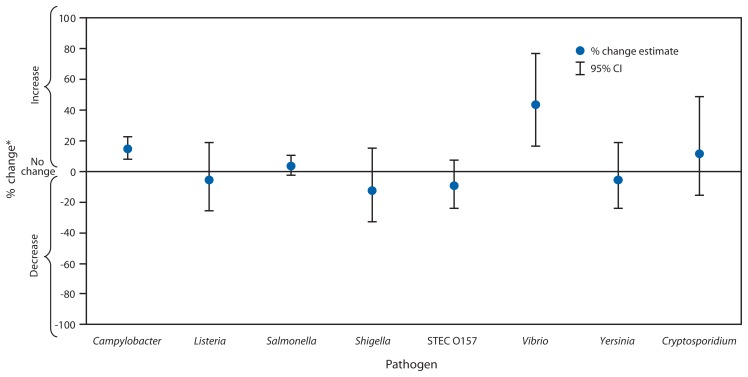
Estimated percentage change in incidence of laboratory-confirmed bacterial and parasitic infections in 2012 compared with average annual incidence during 2006–2008, by pathogen — Foodborne Diseases Active Surveillance Network, United States **Abbreviations:** CI = confidence interval; STEC = Shiga toxin–producing *Escherichia coli*. ^*^ *No significant change* = 95% CI is both above and below the no change line; *significant increase* = estimate and entire CI are above the no change line; *significant decrease* = estimate and entire CI are below the no change line.

**FIGURE 2:**
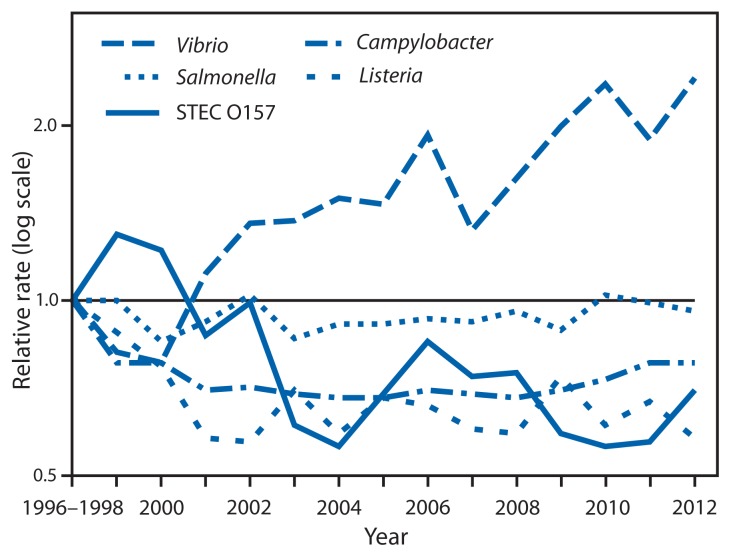
Relative rates of laboratory-confirmed infections with *Campylobacter*, STEC^*^ O157, *Listeria*, *Salmonella*, and *Vibrio* compared with 1996–1998 rates, by year — Foodborne Diseases Active Surveillance Network, United States, 1996–2012^†^ ^*^ Shiga toxin–producing *Escherichia coli*. ^†^ The position of each line indicates the relative change in the incidence of that pathogen compared with 1996–1998. The actual incidences of these infections cannot be determined from this figure.

The estimated incidence of infection was higher in 2012 compared with 2006–2008 for *Campylobacter* (14% increase; confidence interval [CI]: 7%–21%) and *Vibrio* (43% increase; CI: 16%–76%) and unchanged for other pathogens (Figure 1). In comparison with 1996–1998, incidence of infection was significantly lower for *Campylobacter*, *Listeria*, *Shigella*, STEC O157, and *Yersinia*, whereas the incidence of *Vibrio* infection was higher (Figure 2). The overall incidence of infection with six key pathogens** transmitted commonly through food was lower in 2012 (22% decrease; CI: 11%–32%) compared with 1996–1998 and unchanged compared with 2006–2008.

The incidence of infections with specific *Salmonella* serotypes in 2012, compared with 2006–2008, was lower for Typhimurium (19% decrease; CI: 10%–28%), higher for Newport (23% increase; CI: 1%–50%), and unchanged for Enteritidis. Compared with 1996–1998, the incidence of infection was significantly higher for Enteritidis and Newport, and lower for Typhimurium.

Among 63 cases of postdiarrheal HUS in children aged <18 years (0.57 cases per 100,000 children) in 2011, 33 (52%) occurred in children aged <5 years (1.09 cases per 100,000). Compared with 2006–2008, the incidence was significantly lower for children aged <5 years (44% decrease; CI: 18%–62%) and for children aged <18 years (29% decrease; CI: 4%–47%).

## Hospitalizations and Deaths

In 2012, FoodNet identified 4,563 hospitalizations and 68 deaths among cases of infection with pathogens transmitted commonly through food (Table 1). The percentage of patients hospitalized ranged from 15% for *Campylobacter* to 96% for *Listeria* infections. The percentage hospitalized was greatest among those aged ≥65 years for STEC O157 (67%), *Vibrio* (58%), *Salmonella* (55%), *Cyclospora* (50%), *Shigella* (41%), STEC non-O157 (34%), *Cryptosporidium* (33%), and *Campylobacter* (31%). At least 95% of patients with *Listeria* infection in each age group†† with cases were hospitalized. The percentage of patients who died ranged from 0% for *Yersinia* and *Cyclospora* to 11% for *Listeria* infections. The percentage that died was highest among persons aged ≥65 years for *Vibrio* (6%), *Salmonella* (2%), STEC O157 (2%), *Cryptosporidium* (1%), *Shigella* (1%), and *Campylobacter* (0.2%).

### Editorial Note

In 2012, the incidence of infections caused by *Campylobacter* and *Vibrio* increased from the 2006–2008 period, whereas the incidence of infections caused by *Cryptosporidium*, *Listeria*, *Salmonella*, *Shigella*, STEC O157, and *Yersinia* was unchanged. These findings highlight the need to continue to identify and address food safety gaps that can be targeted for action by the food industry and regulatory authorities.

After substantial declines in the early years of FoodNet surveillance, the incidence of *Campylobacter* infection has increased to its highest level since 2000. *Campylobacter* infections are more common in the western U.S. states and among children aged <5 years (3). Although most infections are self-limited, sequelae include reactive arthritis and Guillain-Barré syndrome.§§ Associated exposures include consumption of poultry, raw milk, produce, and untreated water, and animal contact (4,5).

Declines in U.S. campylobacteriosis during 1996–2001 might have been related to measures meat and poultry processors implemented to comply with the Pathogen Reduction and Hazard Analysis and Critical Control Points (HACCP) systems regulations issued by USDA-FSIS in the late 1990s.¶¶ In 2011, USDA-FSIS issued new *Campylobacter* performance standards for U.S. chicken and turkey processors.*** Continued FoodNet surveillance can help to assess the public health impact of these standards and other changes. Detailed patient exposure information coupled with information on strain subtypes could help in assessing the relative contribution of various sources of infection and the effectiveness of control measures.

Although a significant increase was observed in reported *Vibrio* infections, the number of such infections remains low (6). *Vibrios* live naturally in marine and estuarine waters, and many infections are acquired by eating raw oysters (7). These infections are most common during warmer months, when waters contain more *Vibrio* organisms. Infections can be prevented by postharvest treatment of oysters with heat, freezing, or high pressure (8), or by thorough cooking. Persons who are immunocompromised or have impaired liver function should be informed that consuming raw seafood carries a risk for severe *Vibrio* infection. *Vibrios* also cause wound and soft-tissue infections among persons who have contact with water; for example, *Vibrio alginolyticus* typically causes ear infection (9).

The decrease in incidence of HUS in 2011 compared with 2006–2008 mirrors the decrease in the incidence of STEC O157 infection observed in 2011. The incidence of STEC O157 infection, which had declined since 2006, was no longer decreasing in 2012, and now exceeds the previously met *Healthy People 2010* target of one case per 100,000 persons. The continued increase in STEC non-O157 infections likely reflects increasing use by clinical laboratories of tests that detect these infections.

What is already known on this topic?

The incidence of infections transmitted commonly by food that are tracked by the Foodborne Diseases Active Surveillance Network (FoodNet) has changed little in recent years. Foodborne illness continues to be an important public health problem.

What is added by this report?

Preliminary surveillance data show that the incidence of infections caused by *Campylobacter* and *Vibrio* increased in 2012, whereas incidence of other foodborne infections tracked by FoodNet was unchanged (i.e., *Cryptosporidium*, *Listeria*, *Salmonella*, *Shigella*, Shiga toxin–producing *Escherichia coli* O157, and *Yersinia*).

What are the implications for public health practice?

Reducing the incidence of foodborne infections will require commitment and action to implement measures known to reduce contamination of food and to develop new measures. Farmers, the food industry, regulatory agencies, the food service industry, consumers, and public health authorities all have a role.

FoodNet surveillance relies on isolation of bacterial pathogens by culture of clinical specimens; therefore, the increasing use of culture-independent tests for *Campylobacter* and STEC might affect the reported incidence of infection (10). Data on persons with only culture-independent evidence of infection suggests that in 2012, the number of laboratory-identified *Campylobacter* cases could have been 9% greater and the number of STEC (O157 and non-O157) cases 7%–19% greater than that reported (CDC, unpublished data, 2013). The lack of recent decline in STEC O157 incidence is of concern; continued monitoring of trends in the incidence of HUS and use of culture-independent testing might aid in interpreting future data on STEC O157 incidence.

The findings in this report are subject to at least four limitations. First, health-care–seeking behaviors and other characteristics of the population in the surveillance area might affect the generalizability of the findings. Second, many infections transmitted commonly through food (e.g., norovirus infection) are not monitored by FoodNet because these pathogens are not identified routinely in clinical laboratories. Third, the proportion of illnesses transmitted by nonfood routes differs by pathogen, and the route cannot be determined for individual, nonoutbreak-associated illnesses and, therefore, the data provided in this report do not exclusively relate to infections from foodborne sources. Finally, in some cases counted as fatal, the infection with the enteric pathogen might not have been the primary cause of death.

Most foodborne illnesses can be prevented. Progress has been made in decreasing contamination of some foods and reducing illness caused by some pathogens, as evidenced by decreases in earlier years. In 2010, FDA passed the Egg Safety Rule,††† designed to decrease contamination of shell eggs with *Salmonella* serotype Enteritidis. In 2011, USDA-FSIS tightened its performance standard for *Salmonella* contamination to a 7.5% positive rate for whole broiler chickens.§§§ Finally, the Food Safety Modernization Act of 2011 gives FDA additional authority to improve food safety and requires CDC to strengthen surveillance and outbreak response.¶¶¶ Collection of comprehensive surveillance information further supports reductions in foodborne infections by helping to determine where to target prevention efforts, supporting efforts to attribute infections to sources, guiding implementation of measures known to reduce food contamination, and informing development of new measures. Because consumers can bring an added measure of safety during food storage, handling, and preparation, they are advised to seek out food safety information, which is available online.****

## Figures and Tables

**TABLE 1:** Number of cases of bacterial and parasitic infection, hospitalizations, and deaths, by pathogen — Foodborne Diseases Active Surveillance Network, United States, 2012*

	Cases			Hospitalizations		Deaths	
			
Pathogen	No.	Incidence†	Objective§	No.	(%)	No.	(%)
**Bacteria**							
*Campylobacter*	6,793	14.30	8.5	1,044	(15)	6	(0.09)
*Listeria*	121	0.25	0.2	116	(96)	13	(10.74)
*Salmonella*	7,800	16.42	11.4	2,284	(29)	33	(0.42)
*Shigella*	2,138	4.50	N/A¶	491	(23)	2	(0.09)
STEC O157	531	1.12	0.6	187	(35)	1	(0.19)
STEC non-O157	551	1.16	N/A	88	(16)	1	(0.18)
*Vibrio*	193	0.41	0.2	55	(29)	6	(3.11)
*Yersinia*	155	0.33	0.3	59	(38)	0	(0.00)
**Parasites**							
*Cryptosporidium*	1,234	2.60	N/A	236	(19)	6	(0.49)
*Cyclospora*	15	0.03	N/A	3	(20)	0	(0.00)
**Total**	**19,531**			**4,563**		**68**	

**Abbreviations:** N/A = not available; STEC = Shiga toxin–producing *Escherichia coli*.

* Data for 2012 are preliminary.

† Per 100,000 population.

§ *Healthy People 2020* objective targets for incidence of *Campylobacter*, *Listeria*, *Salmonella*, STEC O157, *Vibrio*, and *Yersinia* infections per 100,000 population.

¶ No national health objective exists for these pathogens.

**TABLE 2:** Incidence* of laboratory-confirmed bacterial and parasitic infections in 2012,† by pathogen and age group — Foodborne Diseases Active Surveillance Network, United States

	Age group (yrs)				
	
Pathogen	<5	5–9	10–19	20–64	≥65
**Bacteria**					
*Campylobacter*	24.08	10.54	9.42	14.54	15.26
*Listeria*	0.17	0.00	0.03	0.17	1.05
*Salmonella*	63.49	19.33	11.26	12.15	17.22
*Shigella*	16.92	14.77	2.96	3.10	1.42
STEC§ O157	4.71	2.31	1.65	0.58	0.74
STEC non-O157	4.81	1.33	1.65	0.70	0.92
*Vibrio*	0.07	0.26	0.14	0.43	0.78
*Yersinia*	1.33	0.29	0.16	0.23	0.49
**Parasites**					
*Cryptosporidium*	3.68	3.09	1.70	2.54	3.01
*Cyclospora*	0.00	0.00	0.00	0.04	0.03

* Per 100,000 population.

† Data for 2012 are preliminary.

§ Shiga toxin–producing *Escherichia coli.*

## Footnotes

* Additional information available at http://www.cdc.gov/foodnet.

† The overall incidence of infection combines data for *Campylobacter*, *Listeria*, *Salmonella*, STEC O157, *Vibrio*, and *Yersinia*, six key bacterial pathogens for which >50% of illnesses are estimated to be transmitted by food.

§ FoodNet personnel regularly contact clinical laboratories to ascertain all laboratory-confirmed infections in residents of the surveillance areas.

¶ Final incidence rates will be reported when population estimates for 2012 are available.

** *Campylobacter*, *Listeria*, *Salmonella*, STEC O157, *Vibrio*, and *Yersinia*.

†† Age groups defined as <5 years, 5–9 years, 10–19 years, 20–64 years, and ≥65 years.

§§ Additional information available at http://www.who.int/mediacentre/factsheets/fs255/en/index.html.

¶¶ Additional information available at http://www.fsis.usda.gov/oppde/rdad/frpubs/93-016f.pdf.

*** Additional information is available at http://www.fsis.usda.gov/science/haccp_verification_campylobacter_results_2011/index.asp.

††† Additional information available at http://www.fda.gov/food/guidanceregulation/guidancedocumentsregulatoryinformation/eggs/ucm170615.htm.

§§§ Additional information available at http://www.gpo.gov/fdsys/pkg/FR-2011-03-21/pdf/2011-6585.pdf.

¶¶¶ Additional information available at http://www.fda.gov/food/guidanceregulation/fsma/ucm242500.htm.

**** Additional food safety information is available at http://www.cdc.gov/winnablebattles/foodsafety/index.html, http://www.foodsafety.gov and http://www.fightbac.org.
